# Anticoagulation Prescription and Outcomes in Relation to Renal Function in Patients with Atrial Fibrillation: Results from GLORIA-AF

**DOI:** 10.1055/s-0040-1722706

**Published:** 2021-02-06

**Authors:** Sake J. van der Wall, Christine Teutsch, Sergio J. Dubner, Hans-Christoph Diener, Jonathan L. Halperin, Chang Sheng Ma, Kenneth J. Rothman, Miney Paquette, Kristina Zint, Lionel Riou França, Shihai Lu, Gregory Y. H. Lip, Menno V. Huisman, on behalf of the GLORIA-AF Investigators, Dzifa Abban, Nasser Abdul, Mark Abelson, Alan Ackermann, Fran Adams, Luthando Adams, Pedro Adragão, Walter Ageno, Rajesh Aggarwal, Sergio Agosti, Javier Aguila Marin, Francisco Aguilar, Julio Alberto Aguilar Linares, Luis Aguinaga, Zia Ahmad, Paul Ainsworth, Kamal Al Ghalayini, Saad Al Ismail, Abdelfatah Alasfar, Abdul Alawwa, Raed Al-Dallow, Lisa Alderson, Dimitrios Alexopoulos, Abdullah Ali, Malik Ali, Pareed Aliyar, Tammam Al-Joundi, Soufian Al Mahameed, Hossein Almassi, Khalid Almuti, Mohamed Al-Obaidi, Mohamed Alshehri, Ute Altmann, Alvaro Rabelo Alves, Ayham Al-Zoebi, Walid Amara, Mathieu Amelot, Nima Amjadi, Fabrizio Ammirati, Nabil Andrawis, Denis Angoulvant, Giorgio Annoni, Gerardo Ansalone, Sorin Alexandru Antonescu, Mehrdad Ariani, Juan Carlos Arias, Sébastien Armero, Rohit Arora, Chander Arora, William Ashcraft, M. Shakil Aslam, Alfredo Astesiano, Philippe Audouin, Charles Augenbraun, S. Aydin, Rabih Azar, Abul Azim, Shahid Aziz, Luciano Marcelo Backes, Mirza Baig, Suchdeep Bains, Asaad Bakbak, Seth Baker, Karim Bakhtiar, Richard Bala, Jonathan Banayan, Stellan Bandh, Shigenobu Bando, Subhash Banerjee, Alan Bank, Olga Barbarash, Gonzalo Barón, Craig Barr, Carlos Barrera, John Barton, Vanja Basic Kes, Giovanni Baula, Hamid Bayeh, Nooshin Bazargani, Steffen Behrens, Alan Bell, Juan Benezet-Mazuecos, Bouziane Benhalima, Philippe Berdagué, B. J. Berg van den, P. F.M.M. Bergen van, Edvard Berngard, Richard Bernstein, Percy Berrospi, Sergio Berti, Vicente Bertomeu, Andrea Berz, Paulo Bettencourt, Robert Betzu, Jan Beyer-Westendorf, Ravi Bhagwat, Toby Black, Jorge Hugo Blanco Ibaceta, Stephen Bloom, Edwin Blumberg, Mario Bo, Valerie Bockisch, Ellen Bøhmer, Maria Grazia Bongiorni, Giuseppe Boriani, Ralph Bosch, D. J. Boswijk, Jochen Bott, Edo Bottacchi, Marica Bracic Kalan, Axel Brandes, Bjørn Bratland, Donald Brautigam, Nicolas Breton, P. J.A.M. Brouwers, Kevin Browne, Jordi Bruguera, Myriam Brunehaut, Claude Brunschwig, Hervé Buathier, Aurélie Buhl, John Bullinga, Kenneth Butcher, Jose Walter Cabrera Honorio, Alberto Caccavo, Didier Cadinot, Shanglang Cai, Valeria Calvi, John Camm, Rui Candeias, James Capo, Alessandro Capucci, Juliano Novaes Cardoso, Yan Carlos Duarte Vera, Brian Carlson, Paula Carvalho, Susanna Cary, Rene Casanova, Gavino Casu, Simon Cattan, Claudio Cavallini, Guillaume Cayla, Tae Joon Cha, Kwang Soo Cha, Said Chaaban, Jei Keon Chae, Krishnan Challappa, Sunil Chand, Harinath Chandrashekar, Mark Chang, Paul Charbel, Ludovic Chartier, Kausik Chatterjee, Aamir Cheema, Shih-Ann Chen, Pierre Chevallereau, Fu-Tien Chiang, Francesco Chiarella, Lin Chih-Chan, Yong Keun Cho, Dong Ju Choi, Guy Chouinard, Danny Hoi Fan Chow, Dimitrios Chrysos, Galina Chumakova, Eduardo Julián José Roberto Chuquiure Valenzuela, Tomas Cieza-Lara, Violeta Cindea Nica, Vlad Ciobotaru, David Cislowski, Olivier Citerne, Matthias Claus, Anthony Clay, Piers Clifford, Serge Cohen, Andrew Cohen, Furio Colivicchi, Rónán Collins, Steve Compton, Sean Connors, Alberto Conti, Gabriel Contreras Buenostro, Gregg Coodley, Martin Cooper, Lynn Corbett, Oran Corey, Julián Coronel, John Corrigan, Rosa Ysabel Cotrina Pereyra, Yves Cottin, Benoit Coutu, Aurel Cracan, Peter Crean, James Crenshaw, H. J.G.M. Crijns, Charles Crump, Fred Cucher, David Cudmore, Lianqun Cui, John Culp, Harald Darius, Patrick Dary, Olivier Dascotte, Ira Dauber, Thomas Davee, Ruth Davies, Gershan Davis, Jean-Marc Davy, Mark Dayer, Axel De La Briolle, Manuel de Mora, Eduardo De Teresa, Luc De Wolf, Eric Decoulx, Sasalu Deepak, Pascal Defaye, Freddy Del-Carpio Munoz, Diana Delic Brkljacic, Laurent Deluche, Sylvain Destrac, N. Joseph Deumite, Silvia Di Legge, Olivier Dibon, Igor Diemberger, Jean Dillinger, Pedro Dionísio, Stefan Naydenov, Imran Dotani, Elena Dotcheva, Anthony D'Souza, Simon Dubrey, Xavier Ducrocq, Dmitry Dupljakov, Vuong DuThinh, Oscar Pereira Dutra, Dipankar Dutta, Nathalie Duvilla, Johnny Dy, Rainer Dziewas, Charles Eaton, William Eaves, Matthew Ebinger, J. W.M. Eck van, Tim Edwards, Isabel Egocheaga, Clifford Ehrlich, Steven Eisenberg, Abdel El Hallak, Adnan El Jabali, Rami El Mahmoud, Mahfouz El Shahawy, Zayd Eldadah, Fouad Elghelbazouri, Omar Elhag, Mehiar El-Hamdani, Darlene Elias, Adam Ellery, Hassan El-Sayed, A. Elvan, Bernard Erickson, Eric Espaliat, Louis Essandoh, Tamara Everington, Rudolph Evonich, Andrey Ezhov, Lorenzo Fácila, Ramin Farsad, Maxime Fayard, Francesco Fedele, Luis Gustavo Gomes Ferreira, Daniel Ferreira, José Ferreira Santos, Anna Ferrier, Alexandra Finsen, Brian First, Raymond Fisher, John Floyd, Thomas Folk, Catarina Fonseca, Luisa Fonseca, Steven Forman, Magnus Forsgren, Malcolm Foster, Nathan Foster, Michael Frais, Brad Frandsen, Thierry Frappé, Ramon Freixa, William French, Marina Freydlin, Siegfried Frickel, Ana Gabriela Fruntelata, Shigeru Fujii, Yusuke Fujino, Hiroshi Fukunaga, Yutaka Furukawa, Matthias Gabelmann, Michael Gabris, Niels Gadsbøll, Pavel Galin, Michel Galinier, Ricky Ganim, Ronnie Garcia, Antonio Garcia Quintana, Olivier Gartenlaub, Conrad Genz, Frédéric Georger, Jean-Louis Georges, Steven Georgeson, Ali Ghanbasha, Evaldas Giedrimas, Mariusz Gierba, Eve Gillespie, Alberto Giniger, Alexandros Gkotsis, Joachim Gmehling, Jacek Gniot, Peter Goethals, Ronald Goldberg, Britta Goldmann, David Goldscher, Sergey Golitsyn, Efrain Alonso Gomez Lopez, Juan Esteban Gomez Mesa, Efrain Gonzalez, Emilio Gonzalez Cocina, Carlos Gonzalez Juanatey, Vladimir Gorbunov, Brian Gordon, Hervé Gorka, Charles Gornick, Diana Gorog, Franz Goss, Andreas Götte, Pascal Goube, Ioannis Goudevenos, Dudley Goulden, Brett Graham, Angel Grande, Cesare Greco, Martin Green, Gerald Greer, Uwe Gremmler, Paul Grena, Yuriy Grinshstein, Martin Grond, Edoardo Gronda, Francois Grondin, Gerian Grönefeld, J. R. Groot de, Gabriele Guardigli, Thomas Guarnieri, Carolina Guevara Caiedo, Alexandre Guignier, Michele Gulizia, Michael Gumbley, Dhiraj Gupta, Terrence Hack, Winfried Haerer, Joseph Hakas, Christian Hall, James Hampsey, Georgios Hananis, Basel Hanbali, Franklin Handel, Joe Hargrove, David Hargroves, Kenneth Harris, David Hartley, Tetsuya Haruna, Yoshiki Hata, Emil Hayek, Jeff Healey, Steven Hearne, Geir Heggelund, M. E.W. Hemels, Yann Hemery, Sam Henein, Benhur Henz, Sung-Ho Her, Paul Hermany, Mauro Esteves Hernandes, Yorihiko Higashino, Michael Hill, Tetsuo Hisadome, Eiji Hishida, James Hitchcock, Etienne Hoffer, Matthew Hoghton, Clare Holmes, Suk Keun Hong, Marie-Paule Houppe Nousse, Victor Howard, Li Fern Hsu, Chi-Hung Huang, David Huckins, Kier Huehnergarth, A. Huizenga, Richard Huntley, Gamal Hussein, Gyo-Seung Hwang, Oyidie Igbokidi, Ignacio Iglesias, Margaret Ikpoh, Davide Imberti, Hüseyin Ince, Ciro Indolfi, Tatiana Ionova, John Ip, Didier Irles, Harukazu Iseki, Younus Ismail, Noah Israel, Steven Isserman, Bruce Iteld, Galina Ivanchura, Ramakrishnan Iyer, Venkat Iyer, Ruben Omar Iza Villanueva, Ewart Jackson-Voyzey, Naseem Jaffrani, Frank Jäger, Manish Jain, Martin James, Yann Jamon, Sung Won Jang, Cesar Augusto Pereira Jardim, Nabil Jarmukli, Robert Jeanfreau, Ronald Jenkins, Xianyan Jiang, Heng Jiang, Tiemin Jiang, Nan Jiang, Javier Jimenez, Robert Jobe, Ian Joffe, Bengt Johansson, Nick Jones, Jose Carlos Moura Jorge, Bernard Jouve, Mayar Jundi, Werner Jung, Byung Chun Jung, Kyung Tae Jung, Samer Kabbani, Ameer Kabour, Chrystalenia Kafkala, Koji Kajiwara, Larisa Kalinina, Priit Kampus, Junji Kanda, Shaival Kapadia, Amin Karim, Laszlo Karolyi, Hisham Kashou, Andreas Kastrup, Apostolos Katsivas, Elizabeth Kaufman, Kazuya Kawai, Kenji Kawajiri, John Kazmierski, Phil Keeling, Galal Ali Kerfes, José Francisco Kerr Saraiva, Galina Ketova, Ajit Khaira, Muhammad Khalid, Elena Khludeeva, Aleksey Khripun, Doo Il Kim, Dae Kyeong Kim, Nam Ho Kim, Ki Seok Kim, Young-Hoon Kim, Jin bae Kim, June Soo Kim, Jeong Su Kim, Elena Kinova, Alexander Klein, Christoph Kleinschnitz, James Kmetzo, G. Larsen Kneller, Aleksandar Knezevic, Stanley Koch, Kai Koenig, Su Mei Angela Koh, Martin Köhrmann, Jay Koons, Ravikiran Korabathina, Olga Korennova, Martin Koschutnik, Edward Kosinski, Dragan Kovacic, Jacek Kowalczyk, Natalya Koziolova, J. A. Kragten, Lars Udo Krause, Imad Kreidieh, B. J. Krenning, Kannappan Krishnaswamy, Waldemar Krysiak, Karl-Heinz Kuck, Somnath Kumar, Thomas Kümler, Malte Kuniss, Jen-Yuan Kuo, Achim Küppers, Karla Kurrelmeyer, Tak Kwan, Eisho Kyo, Arthur Labovitz, Alain Lacroix, Andy Lam, Fernando Tomas Lanas Zanetti, Charles Landau, Giancarlo Landini, Wilfried Lang, Torben Bjerregaard Larsen, Volker Laske, Karine Lavandier, Nicki Law, Moon Hyoung Lee, Daniel Lee, Ana Leitão, Dominique Lejay, Malgorzata Lelonek, Radoslaw Lenarczyk, Patrick Leprince, Benoit Lequeux, Matthias Leschke, Nicolas Ley, Zicheng Li, Yansheng Li, Xiaodong Li, Zhanquan Li, Weihua Li, Jianqiu Liang, Ira Lieber, Michael Lillestol, Ramon Horacio Limon Rodriguez, Hailong Lin, Gregory Lip, Jennifer Litchfield, Zhitao Liu, Xuebo Liu, Yalin Liu, Feng Liu, Wenhui Liu, Guillermo Antonio Llamas Esperon, Jose Luis Llisterri, Ted Lo, Eric Lo, Jose Maria Lobos, Bernhard-Paul Lodde, Philippe Loiselet, José López-Sendón, Adalberto Menezes Lorga Filho, Ido Lori, Ming Luo, Steven Lupovitch, Philippe Lyrer, Hamed M. Zuhairy, Changsheng Ma, Genshan Ma, Hong Ma, Irene Madariaga, Koji Maeno, Dominique Magnin, Shahid Mahmood, Karen Mahood, Gustavo Maid, Sumeet Mainigi, Konstantinos Makaritsis, José Arturo Maldonado Villalon, Rohit Malhotra, Amir Malik, Catherine Mallecourt, Rajiv Mallik, Rickey Manning, Athanasios Manolis, Ioannis Mantas, Fernando Gabriel Manzur Jattin, Niccolo' Marcionni, Francisco Marín, Antonio Martín Santana, Jorge Martinez, Luis Martinez, Petra Maskova, Norberto Matadamas Hernández, Simon Matskeplishvili, Katsuhiro Matsuda, Alenka Mavri, Erik May, Nolan Mayer, Pilar Mazon, John McClure, Terry McCormack, William McGarity, Michael McGuire, Hugh McIntyre, Paul McLaughlin, Brent McLaurin, Feliz Alvaro Medina Palomino, Paresh Mehta, Reza Mehzad, Andreas Meinel, Francesco Melandri, Amparo Mena, Hirosi Meno, Dhananjai Menzies, Kneale Metcalf, Beat Meyer, Jacek Miarka, Frank Mibach, Dominik Michalski, Patrik Michel, Rami Mihail Chreih, Ghiath Mikdadi, Magdy Mikhail, Milan Mikus, Davor Milicic, Constantin Militaru, Gary Miller, Christos Milonas, Bogdan Minescu, Iveta Mintale, Aurélien Miralles, Tristan Mirault, Dinesh Mistry, George Mitchell, Nicoleta Violeta Miu, Naomasa Miyamoto, Tiziano Moccetti, Akber Mohammed, Azlisham Mohd Nor, Dora Ines Molina de Salazar, Giulio Molon, David Molony, Sergio Mondillo, Lluis Mont, Rajendra Moodley, Roger Moore, Dalmo Antonio Ribeiro Moreira, Kiyoo Mori, Andrew Moriarty, Jacek Morka, Nikitas Moschos, Marco Antônio Mota Gomes, Nicolas Mousallem, Angel Moya, Andreas Mügge, Thomas Mulhearn, Jean-Joseph Muller, Carmen Manuela Muresan, Derek Muse, Wlodzimierz Musial, Francesco Musumeci, Venkatesh Nadar, Thuraia Nageh, Priya Nair, Hidemitsu Nakagawa, Yuichiro Nakamura, Toru Nakayama, Ki-Byeong Nam, Dmitry Napalkov, Indira Natarajan, Hemal Nayak, Libor Nechvatal, James Neiman, Pamela Nerheim, Fernando Carvalho Neuenschwander, Kunihiro Nishida, Alexey Nizov, Tatiana Novikova, Salvatore Novo, Ewa Nowalany-Kozielska, Emmanuel Nsah, Juan Carlos Nunez Fragoso, Ole Nyvad, Manuel Odin de Los Rios Ibarra, Martin O'Donnell, Philip O'Donnell, Dong Jin Oh, Yong Seog Oh, Chia Theng Daniel Oh, Gilles O'Hara, Kostas Oikonomou, Juan Jose Olalla, Zoran Olivari, Richard Oliver, Christoforos Olympios, John Osborne, Joaquin Osca, Raed Osman, Abayomi Osunkoya, Benzy Padanilam, Elizaveta Panchenko, A. Shekhar Pandey, Angelo Amato Vicenzo de Paola, Alexander Paraschos, Herbert Pardell, Hyung Wook Park, Jong Sung Park, Ratika Parkash, Ian Parker, Eric Parrens, Robert Parris, Enrico Passamonti, Jaydutt Patel, Rajesh Patel, William H. Pentz, Viktor Persic, Francesco Perticone, Patrick Peters, Sanjiv Petkar, Luis Felipe Pezo, David Pham, Gérald Phan Cao Phai, Stephen Phlaum, Julien Pineau, Armando Pineda-Velez, Riccardo Pini, Arnold Pinter, Fausto Pinto, Salvatore Pirelli, Nediljko Pivac, Attilia Maria Pizzini, Darko Pocanic, Cristian Gheorghe Calin Podoleanu, Carisi Anne Polanczyk, Petr Polasek, Zdravka Poljakovic, Stewart Pollock, Jose Polo, James Poock, Holger Poppert, Yamile Porro, Antonio Pose, François Poulain, Jean-Ernst Poulard, Joe Pouzar, Petr Povolny, Domingo Pozzer, Athanasios Pras, Neeraj Prasad, Sébastien Prevot, Konstantin Protasov, Laurent Prunier, John Puleo, Maurice Pye, Fatma Qaddoura, Jean-Michel Quedillac, Dimitar Raev, Sidiqullah Rahimi, Arturo Raisaro, Bhola Rama, Nandkishore Ranadive, Katie Randall, Naresh Ranjith, Nuno Raposo, Haroon Rashid, Christa Raters, Ursula Rauch-Kroehnert, Thomas Rebane, Stefan Regner, Michael Renzi, Miguel Agustin Reyes Rocha, Shabbir Reza, Luigi Ria, Dimitrios Richter, Hans Rickli, Kyle Rickner, Werner Rieker, Fausto Rigo, Tomas Ripoll, Luiz Eduardo Fonteles Ritt, Douglas Roberts, Carlos Rodríguez Pascual, Ignacio Rodriguez Briones, Humberto Rodriguez Reyes, Marc Roelke, Mark Roman, Francesco Romeo, E. Ronner, Thomas Ronziere, F. A. Rooyer, David Rosenbaum, Sherryn Roth, Nadezda Rozkova, Miroslav Rubacek, Frank Rubalcava, Olesya Rubanenko, Andrew Rubin, Mariano Ruiz Borret, Karin Rybak, Hani Sabbour, Oscar Saenz Morales, Tetsuo Sakai, Abraham Salacata, Ilsbe Salecker, Adrien Salem, Marwan Salfity, Rafael Salguero, Alessandro Salvioni, Mercedes Samson, Gregorio Sanchez, Chirag Sandesara, Wladmir Faustino Saporito, Taishi Sasaoka, Payman Sattar, Daniel Savard, Pierre-Jean Scala, Jacques Scemama, Thierry Schaupp, Peter Schellinger, Carlos Scherr, Karl-Heinz Schmitz, Bettina Schmitz, Lisa Schmitz, Robert Schnitzler, Steffen Schnupp, Peter Schoeniger, Norbert Schön, Stefan Schuster, Peter Schwimmbeck, Clare Seamark, Ruediger Seebass, Karl-Heinz Seidl, Barry Seidman, Jaroslaw Sek, Lakshmanan Sekaran, Yoshinori Seko, Pablo Andres Sepulveda Varela, Begoña Sevilla, Vinay Shah, Anil Shah, Neerav Shah, Aman Shah, Jeffrey Shanes, Ali Sharareh, Vijay Kumar Sharma, Louise Shaw, Yutaka Shimizu, Hideki Shimomura, Dong Gu Shin, Eun-Seok Shin, Junya Shite, Mohammad Shoukfeh, Charles Shoultz, Frank Silver, Iveta Sime, T. A. Simmers, Dinesh Singal, Narendra Singh, Peter Siostrzonek, Mohiburrahman Sirajuddin, Mika Skeppholm, Didier Smadja, Richard Smith, David Smith, Hassan Soda, C. Wilson Sofley, Adam Sokal, Rodolfo Sotolongo, Olga Ferreira de Souza, Jon Arne Sparby, Jindrich Spinar, David Sprigings, Alex Spyropoulos, Dimitrios Stakos, Alon Steinberg, Clemens Steinwender, Georgios Stergiou, H. William Stites, Anastas Stoikov, Ruth Strasser, Witold Streb, Ioannis Styliadis, Guohai Su, Xi Su, Rafael Martin Suarez, Wanda Sudnik, Atsushi Sueyoshi, Kai Sukles, Li Sun, Randeep Suneja, Peter Svensson, Antonius Ziekenhuis, Janko Szavits-Nossan, Jens Taggeselle, Yuichiro Takagi, Amrit Takhar, Julio Tallet, Angelika Tamm, Shozo Tanaka, Katsumi Tanaka, Aylmer Tang, Sherman Tang, Tiziana Tassinari, Shinji Tayama, Muzahir Tayebjee, Ulrich Tebbe, Jose Teixeira, Dan Nicolae Tesloianu, Pascal Tessier, S. H.K. The, Jérome Thevenin, Harold Thomas, Serge Timsit, Robert Topkis, Mikhail Torosoff, Emmanuel Touze, Thalie Traissac, Elina Trendafilova, Barry Troyan, Wenchi Kevin Tsai, Hung Fat Tse, Hiroshi Tsutsui, Takashi Tsutsui, Y. S. Tuininga, Minang Turakhia, Samir Turk, Wayne Turner, Arnljot Tveit, Shannon Twiddy, Richard Tytus, Gerald Ukrainski, Salvador Bruno Valdovinos Chavez, Eric Van De Graaff, Peter Vanacker, Panagiotis Vardas, Michael Vargas, Vassilios Vassilikos, Juan Vazquez, Asok Venkataraman, Paolo Verdecchia, Ernst Günter Vester, Hubert Vial, Dragos Vinereanu, Anthony Vlastaris, Craig Vogel, Jürgen vom Dahl, Matthias von Mering, Kishor Vora, Paul Wakefield, Jasjit Walia, Thomas Walter, Mingsheng Wang, Ningfu Wang, Feng Wang, Xinhua Wang, Zulu Wang, Kuo-Yang Wang, Kouki Watanabe, Jeanne Wei, Christian Weimar, Renate Weinrich, Ming-Shien Wen, Kevin Wheelan, Jens Wicke, Marcus Wiemer, Beate Wild, Andreas Wilke, Stephan Willems, Marcus Williams, David Williams, Andreas Winkler, Jost Henner Wirtz, Bernhard Witzenbichler, Danny H K Wong, Ka Sing Lawrence Wong, Brian Wong, Beata Wozakowska-Kaplon, Zhaohui Wu, Shulin Wu, Nell Wyatt, Yong Xu, Xiangdong Xu, Akira Yamada, Kazuya Yamamoto, Hiroki Yamanoue, Takeshi Yamashita, Ping Yen Bryan Yan, Yanmin Yang, Tianlun Yang, Jing Yao, Chakri Yarlagadda, Kuo-Ho Yeh, Yoto Yotov, Serge Yvorra, Ralf Zahn, José Zamorano, Roberto Zanini, Stuart Zarich, James Zebrack, Sergei Zenin, Elisabeth Louise Zeuthen, Xingwei Zhang, Quansan Zhang, Dadong Zhang, Donghui Zhang, Huanyi Zhang, Shuiping Zhao, Xinwen Zhao, Yang Zheng, Qiangsun Zheng, Jing Zhou, Jian Zhou, Sergio Luiz Zimmermann, Rainer Zimmermann, L. Steven Zukerman, C. Zwaan van der

**Affiliations:** 1 Department of Thrombosis and Hemostasis, Leiden University Medical Center, Leiden, The Netherlands; 2 Department of CardioMetabolism and Respiratory Medicine, Boehringer Ingelheim International GmbH, Ingelheim, Germany; 3 Electrophysiology Service, Clínica y Maternidad Suizo Argentina, Buenos Aires, Argentina; 4 Faculty of Medicine, University of Duisburg-Essen, Germany; 5 The Cardiovascular Institute, Icahn School of Medicine at Mount Sinai, New York City, New York, United States; 6 Cardiology Department, Atrial Fibrillation Center, Beijing Anzhen Hospital, Capital Medical University, Beijing, China; 7 RTI Health Solutions, Research Triangle Institute, Research Triangle Park, North Carolina, United States; 8 Department of Medicine, Boehringer Ingelheim, Burlington, Ontario, Canada; Global Epidemiology at Boehringer Ingelheim GmbH, Ingelheim, Germany; 9 Global Epidemiology Department, Boehringer Ingelheim International GmbH, Ingelheim, Germany; 10 Biostatistics and Data Sciences Department, Boehringer Ingelheim Pharmaceuticals, Inc., Ridgefield, Connecticut, United States; 11 Liverpool Centre for Cardiovascular Science, University of Liverpool and Liverpool Heart & Chest Hospital, Liverpool, United Kingdom; 12 Department of Clinical Medicine, Aalborg Thrombosis Research Unit, Aalborg University, Aalborg, Denmark

**Keywords:** atrial fibrillation, anticoagulation, dabigatran, renal function, stroke, bleeding

## Abstract

**Objective** Anticoagulation management in patients with atrial fibrillation (AF) and impaired renal function is challenging. This study aimed to evaluate anticoagulation prescription patterns in relation to renal function and to describe 2-year clinical outcomes among dabigatran users.

**Methods** Global Registry on Long-Term Oral Antithrombotic Treatment in Patients with Atrial Fibrillation (GLORIA-AF) is an international, prospective, and observational study program involving patients with newly diagnosed AF at risk for stroke. Prescription patterns were assessed by creatinine clearance (CrCl) at enrollment. Dabigatran users were followed for 2 years. Clinical outcomes were standardized for stroke and bleeding risk, based on CHA _2_ DS _2_ -VASc and HAS-BLED scores, with missing values imputed.

**Results** Baseline CrCl values were available for 12,056 of 15,308 eligible patients (79%). With declining renal function, prescriptions increased for vitamin K antagonists (VKAs) and decreased for dabigatran (30–47% and 34–12%, respectively). The prescription of other non-vitamin K antagonists remained similar across CrCl groups (14–19%). In 4,873 dabigatran users, standardized stroke rates were low across all CrCl groups; 0.58/100 patient-years (95% confidence interval [CI]: 0.30–0.90) in CrCl ≥80 mL/min, 0.85 (95% CI: 0.48–1.21) in CrCl 50 to 79 mL/min, and 0.33 (95% CI: 0.06–1.11) in CrCl 30 to 49 mL/min. Similarly, major bleeding rates were low and numerically increased with declining renal function (0.68/100 patient-years, 95% CI: 0.39–1.03; 0.92, 95% CI: 0.58–1.32; and 1.26, 95% CI: 0.66–1.97, respectively).

**Conclusion** In patients with AF, VKA prescriptions increased and dabigatran prescriptions decreased with declining renal function. Rates of stroke and major bleeding in dabigatran patients remained low across the categories of renal impairment.

## Introduction

Some degree of renal impairment is estimated to be present in approximately 35% of patients with atrial fibrillation (AF) and is associated with increased risk of both thromboembolic and bleeding events. 1 2 3 Consequently, oral anticoagulation management is challenging and often requires dose adjustment. Suboptimal use of vitamin K antagonists (VKAs) or non-vitamin K antagonists (NOACs) increase the risk of stroke and systemic embolism. 4 5 6 How renal function affects anticoagulation prescription practice is unclear.

All NOACs undergo some degree of renal clearance. Consequently, diminished renal function could lead to drug accumulation and increase the risk of bleeding complications. Clinical practice guidelines recommend caution regarding NOAC use in patients with renal dysfunction. 7 8 Rivaroxaban, apixaban, and edoxaban, but not dabigatran, are approved in Europe for use in patients with severe renal impairment (i.e., a creatinine clearance [CrCl] of <30 mL/min). 8 This differs for the United States, where dabigatran is only contraindicated below a CrCl of 15 mL/min due to the available dose of 75 mg twice daily. 9 Moreover, in the United States, no contraindications exist for rivaroxaban and apixaban based on renal function. Lower doses, however, are recommended for CrCl ≤50 mL/min (rivaroxaban) and serum ≥1.5 mg/dL (apixaban). 10 11

A meta-analysis demonstrated fewer thromboembolic and major bleeding events in patients with mild or moderate renal impairment (CrCL = 30–79 mL/min) treated with NOACs than with VKAs, although there is considerable heterogeneity across studies. 12 Moreover, the effectiveness and safety of NOACs in patients with a CrCl <30 mL/min has not been established.

The purposes of this study were to evaluate anticoagulation prescription patterns in relation to baseline renal function for patients with AF enrolled in phase II of the Global Registry on Long-Term Oral Antithrombotic Treatment in Patients with Atrial Fibrillation (GLORIA-AF) study and to describe clinical outcomes over 2 years in patients who received dabigatran etexilate.

## Methods

### Patients and Study Design

The study design and baseline characteristics of patients enrolled in the GLORIA-AF Registry Program have been described previously. 13 14 In short, GLORIA-AF is an international, prospective, observational and study program, run in three phases, enrolling consecutive adult patients with nonvalvular AF who were newly diagnosed in a variety of clinical settings between 2010 and 2017. The study excluded patients with mechanical heart valves, previous VKA therapy for >60 days, AF due to a generally reversible cause, and life expectancy <1 year. During phase II of the program (patient enrollment from 2011 to 2014), cross-sectional data were collected, regardless of treatment, and patients prescribed dabigatran were followed for up to 2 years, with visits scheduled around 3, 6, 12, and 24 months. Dabigatran was prescribed at the discretion of treating physicians in available doses (150, 110, and 75 mg twice daily, depending on the country and local label). Patients who took at least one dose of dabigatran were included in the outcome event analysis. Final phase II results have been recently been published. 15 In the present report, phase II data were evaluated in relation to baseline renal function. GLORIA-AF was a noninterventional study; hence, prescription requirements were not stipulated in the protocol.

### Study Aim

The aims of the study were to evaluate anticoagulation prescription patterns in patients with different CrCl categories and to describe 2-year incidence rates of stroke, major bleeding, and death for patients treated with dabigatran according to CrCl. The CrCl was calculated according to Cockcroft-Gault formula: CrCl (mL/min) = (140 − age) × weight (kg) × (0.85 if female)/(72 × serum creatinine concentration). Renal impairment was categorized according to international guidelines as no impairment (CrCl ≥ 80 mL/min), mild impairment (CrCl = 50–79 mL/min), moderate impairment (CrCl = 30–49 mL/min), and severe impairment (CrCl < 30 mL/min). 16 17

### Outcomes

Only patients taking dabigatran were followed for the occurrence of clinical outcomes. Stroke was defined as the acute onset of a focal neurologic deficit of presumed vascular origin 24 hours or more or resulting in death. Stroke type was categorized as ischemic, hemorrhagic, or uncertain (based on computed tomography or magnetic resonance scanning, or autopsy). Major bleeding was defined according to criteria of the International Society on Thrombosis and Haemostasis. 18 Deaths were classified as vascular (including bleeding) and nonvascular due to other specified causes (e.g., malignancy) or unknown cause.

### Statistical Analysis

Baseline characteristics of patients were examined by categories of renal function. Continuous variables are presented as the means (±standard deviation [SD]) or medians (with interquartile range [IQR]) and categoric variables as frequency ( *n* ) and percent (%).

For the derivation of incidence rates of dabigatran outcome incidence rates, the risk period began with treatment initiation and ended with permanent dabigatran discontinuation, which was defined by treatment stop +3 days, substitution of another anticoagulant treatment, or study completion. Incidence rates were calculated based on time to first event of interest. Patients prescribed dabigatran who did not take at least one dose ( *n* = 14) were excluded.

To adjust for potential confounding, incidence rates were standardized by HAS-BLED score using two categories, less than 2 or ≥2, and CHA _2_ DS _2_ -VASc scores using three categories, < 3, 3, and ≥4, for a total of six categories when stratifying by both variables. Standardization was accomplished by obtaining a weighted average of the stratum-specific incidence rates, using weights equal to the total number of patient years for all patients in that stratum. Because only a small number of patients had a CrCl <30 mL/min, rates were not reported for that category. HAS-BLED and CrCl had noticeable proportions of patients taking dabigatran with missing data (10 and 23%, respectively); multiple imputation using chained equations was applied to address this. 19 The imputation model was constructed upon 54 clinically relevant baseline patient characteristic variables and the number of imputation is 20. The CIs of the standardized incidence rates were constructed using the bootstrap method. 20

## Results

### Patient Population

Baseline characteristics by CrCl for both the entire population as well as dabigatran users are displayed in Table 1 . Baseline creatinine levels were available for 12,056 of 15,308 eligible patients (79%). For the entire population, a total of 5,116 of 15,308 patients (33%) had CrCl ≥80 mL/min; 4,714 (31%) had CrCl 50 to 79 mL/min; 1,805 (12%) had CrCl 30 to 49 mL/min, 430 (3%) had CrCl <30 mL/min; and 3,243 (21%) had missing values. Patients with a CrCl ≥80 mL/min were younger (63 ± 10 years) than those with CrCl 50 to 79; 30 to 49; or <30 mL/min (74 ± 8, 80 ± 7, 80 ± 11, respectively), or those with unknown CrCl values (71 ± 11 years). Most patients had paroxysmal AF (51–56%, depending on CrCl category), followed by persistent (35–37%) and permanent AF (8–14%). As expected, the mean CHA _2_ DS _2_ -VASc score increased with declining renal function, ranging from 2.6 (SD = 1.3) in patients with a CrCl ≥80 mL/min to 4.7 (1.5) in patients with a CrCl <30 mL/min. A similar trend was observed for the mean HAS-BLED score, ranging from 1.1 (SD = 0.9) to 2.0 (1.0).

**Table 1:** Baseline characteristics of the entire GLORIA-AF population and the subset of patients treated with dabigatran according to creatinine clearance

	Entire GLORIA-AF population ( *n* = 15,308)					Patients treated with dabigatran ( *n* = 4,873)				
Characteristic	CrCl ≥ 80 mL/min ( *n* = 5,116)	CrCl 50–79 mL/min ( *n* = 4,714)	CrCl 30–49 mL/min ( *n* = 1,805)	CrCl < 30 mL/min ( *n* = 430)	Missing CrCl ( *n* = 3,243)	CrCl ≥ 80 mL/min ( *n* = 1,743)	CrCl 50–79 mL/min ( *n* = 1,488)	CrCl 30–49 mL/min ( *n* = 476)	CrCl < 30 mL/min ( *n* = 52)	Missing CrCl ( *n* = 1,114)
Age (y), mean ± SD	63 ± 10	74 ± 8	80 ± 7	80 ± 11	71 ± 11	64 ± 10	74 ± 8	80 ± 7	78 ± 12	71 ± 10
Gender (male), *n* (%)	3290 (64)	2343 (50)	1091 (60)	166 (39)	1827 (56)	1149 (66)	707 (48)	184 (39)	21 (40)	650 (58)
BMI (kg/m ^2^ ), mean ± SD	31 ± 6.7	27 ± 4.7	25 ± 4.6	25 ± 5.2	29 ± 6	32 ± 6.3	27 ± 4.4	25 ± 3.9	25 ± 4.4	29 ± 6
Missing, *n* (%)	7 (0.14)	15 (0.32)	9 (0.50)	0	175 (5.4)	0	3 (0.20)	2 (0.42)	0	43 (3.9)
Type of atrial fibrillation, *n* (%)										
Paroxysmal	2,828 (55)	2,476 (53)	915 (51)	205 (48)	1,735 (54)	922 (53)	762 (51)	234 (49)	23 (44)	587 (53)
Persistent	1,821 (36)	1,719 (37)	632 (35)	166 (39)	1,118 (35)	633 (36)	516 (35)	162 (34)	21 (40)	387 (35)
Permanent	467 (9.1)	519 (11)	258 (14)	59 (14)	390 (12)	188 (11)	210 (14)	80 (17)	8 (15)	140 (13)
CHADS2VASC2 score										
1, *n* (%)	1,237 (24)	366 (7.8)	26 (1.4)	16 (3.7)	485 (15)	345 (20)	82 (5.5)	4 (0.84)	6 (12)	155 (14)
≥ 2, *n* (%)	3,879 (76)	4,348 (92)	1,779 (99)	414 (96)	2,758 (85)	1,398 (80)	1,406 (95)	472 (99)	46 (89)	959 (86)
Mean ± SD	2.6 ± 1.3	3.3 ± 1.7	4.3 ± 1.7	4.7 ± 1.5	3.0 ± 1.4	2.7 ± 1.3	3.6 ± 1.4	4.3 ± 1.4	3.9 ± 1.8	3.1 ± 1.4
HAS-BLED score										
< 3, *n* (%)	4,255 (83)	3,740 (79)	1,351 (75)	287 (67)	2,475 (76)	1,475 (85)	1,240 (83)	378 (79)	39 (75)	903 (81)
≥ 3, *n* (%)	329 (6.4)	506 (11)	263 (15)	107 (25)	191 (5.9)	99 (5.7)	121 (8.1)	48 (10)	10 (19)	52 (4.7)
Mean ± SD	1.1 ± 0.9	1.5 ± 0.8	1.7 ± 0.8	2.0 ± 1.0	1.3 ± 0.8	1.0 ± 0.9	1.4 ± 0.8	1.6 ± 0.8	1.7 ± 0.9	1.2 ± 0.8
Missing, *n* (%)	532 (10)	468 (9.9)	191 (11)	36 (8.4)	577 (18)	169 (10)	127 (8.5)	50 (11)	3 (5.8)	159 (14)
Medications (%)										
Antiplatelet	1,382 (27)	1,234 (26)	536 (30)	124 (29)	798 (25)	274 (16)	245 (17)	89 (19)	11 (21)	159 (14)

Abbreviations: BMI, body mass index; creatinine clearance; SD, standard deviation; TIA, transient ischemic attack; VTE, venous thromboembolism.

Patients treated with dabigatran had baseline characteristics similar to the overall GLORIA-AF population. Of 4,873 patients prescribed dabigatran, 3,759 (77%) had creatinine measurements available at baseline. A total of 1,743 of 4,873 (36%) had CrCl ≥80 mL/min, 1,488 (31%) had CrCl 50 to 79 mL/min, 476 (10%) had CrCl 30 to 49 mL/min, and 52 (1%) had CrCl <30 mL/min.

### Anticoagulation Prescription

**Fig. 1:**
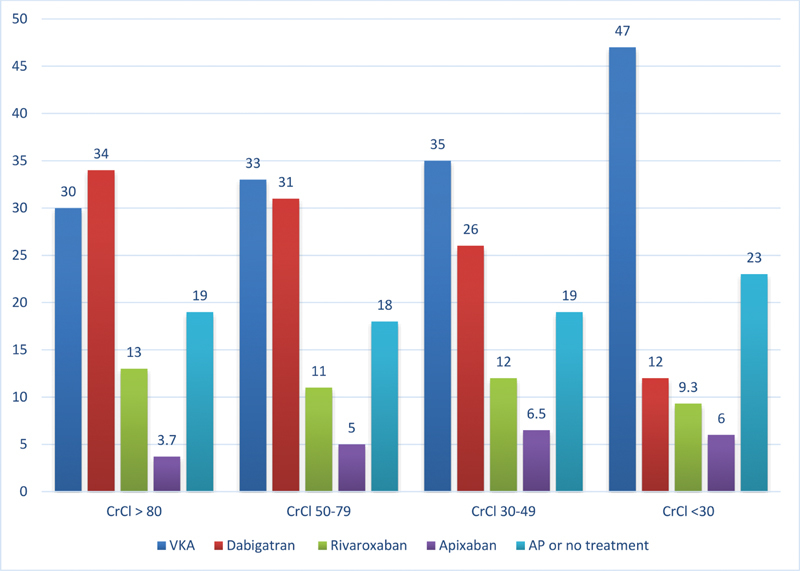
Antithrombotic prescription patterns according to CrCl in GLORIA-AF. Numbers are percentages. CrCl in mL/min. AP, antiplatelet therapy; CrCl, creatinine clearance; VKA, vitamin K antagonist.

At baseline, of all patients with CrCl ≥80 mL/min ( *n* = 5,116), most were prescribed dabigatran ( *n* = 1,743; 34%) or a VKA ( *n* = 1,534; 30%), followed by rivaroxaban ( *n* = 647; 13), antiplatelet therapy ( *n* = 592; 12%), no antithrombotic treatment ( *n* = 389; 8%) and apixaban ( *n* = 188; 3.7%). With declining renal function, prescription rates increased for VKA and decreased for dabigatran (30–47% and 34–12%, respectively; Fig. 1 ). Of the 52 patients taking dabigatran with CrCl <30 mL/min, 41 (79%) were treated with the 150 or 110 mg twice daily dosage, and 11 (21%) received a 75 mg twice-daily dose. Eleven patients receiving the 75 mg twice-daily dose were all located in the United States. Among all dabigatran patients, 0.9% ( *n* = 41) received dabigatran 150 and 110 mg BID and had a CrCl <30 mL/min, constituting a contraindication. Prescription rates for other NOACs were similar across renal function strata (9.3–13% for rivaroxaban and 3.5–6.5% for apixaban, depending on CrCl). As renal function decreased, prescription of other NOAC standard doses decreased and use of low doses increased, although contraindications due to CrCl were not further assessed. The dabigatran standard dose (150 mg twice daily) was prescribed more frequently in patients with normal renal function (72%) than for those with CrCl 50 to 79 (45%) or 30 to 49 mL/min (20%) ( Table 2 ). The prescription rate of antiplatelet or no treatment was consistent across the renal function strata (19–23%, depending on CrCl).

**Table 2:** Anticoagulation dose prescription patterns according to creatinine clearance

Prescribed antithrombotic treatment	CrCl ≥ 80 mL/min ( *n* = 5,116)	CrCl 50–79 mL/min ( *n* = 4,714)	CrCl 30–49 mL/min ( *n* = 1,805)	CrCl < 30 mL/min ( *n* = 430)	Missing CrCl ( *n* = 3,243)	Total ( *n* = 15,308)
VKA	1,534 (30)	1,558 (33)	636 (35)	201 (47)	1,034 (32)	4,963 (32)
Dabigatran						
Total	1,743 (34)	1,488 (31)	476 (26)	52 (12)	1114 (34)	4,873 (32)
150 mg twice daily	1,252 (25)	675 (14)	93 (5.2)	15 (3.5)	629 (19)	2,664 (17)
110 mg twice daily	465 (9.1)	783 (17)	355 (20)	26 (6)	467 (14)	2,105 (14)
75 mg twice daily	18 (0.35)	21 (0.44)	21 (1.2)	11 (2.6)	16 (0.49)	87 (0.57)
Other	8 (0.16)	9 (0.19)	7 (0.38)	0	2 (0.06)	26 (0.17)
Rivaroxaban						
Total	647 (13)	531 (11)	218 (12)	40 (9.3)	319 (9.8)	1,755 (11)
20 mg once daily	590 (12)	388 (8.2)	88 (4.9)	10 (2.3)	236 (7.3)	1,312 (8.6)
15 mg once daily	47 (0.92)	138 (2.9)	128 (7.1)	30 (7.0)	82 (2.5)	425 (2.8)
Other	10 (0.20)	5 (0.11)	2 (0.11)	0	1 (0.031)	18 (0.012)
Apixaban						
Total	188 (3.7)	234 (5.0)	118 (6.5)	26 (6.0)	137 (4.2)	703 (4.6)
5 mg twice daily	180 (3.5)	192 (4.1)	48 (2.7)	6 (1.4)	117 (3.6)	543 (3.5)
2.5 mg twice daily	7 (0.14)	41 (0.87)	69 (3.8)	20 (4.7)	20 (0.62)	157 (1.0)
Other	1 (0.020)	1 (0.021)	1 (0.055)	0	0	3 (0.020)
Other						
Total	1,002 (20)	902 (19)	357 (20)	111 (26)	638 (20)	3,010 (20)
Antiplatelet treatment	613 (12)	543 (12)	242 (13)	63 (15)	379 (12)	1,840 (12)
No antithrombotic treatment	389 (7.6)	359 (7.6)	115 (6.4)	48 (11)	259 (8.0)	1,170 (7.6)

Abbreviations: CrCl, creatinine clearance; NOAC, non-vitamin K oral anticoagulant; VKA, vitamin K antagonist.

Note: All data are reported in numbers (percentages).

### Clinical Outcomes

Clinical outcomes in dabigatran-treated patients are shown in Table 3 . The standardized incidence rates for stroke were 0.58/100 patient-years (95% confidence interval [CI]: 0.30–0.90) for CrCl ≥80 mL/min, 0.85/100 patient-years (95% CI: 0.51–1.2) for CrCl 50 to 79 mL/min, and 0.33/100 patient-years (95% CI: 0.06–1.1) for CrCl 30 to 49 mL/min. The incidence rates for major bleeding were 0.68/100 patient-years (95% CI: 0.39–1.0) for CrCl ≥80 mL/min, 0.92/100 patient-years (95% CI: 0.58–1.3) for CrCl 50 to 79 mL/min, and 1.26 (95% CI: 0.66–2.0) for CrCl 30 to 49 mL/min. The rates for all-cause death were highest in patients with a CrCl 30 to 49 mL/min (4.5/100 patient-years; 95% CI: 3.1–6.3), followed by patients with CrCl 50 to 79 mL/min (2.6/100 patient-years; 95% CI: 2.0–3.3) and patients with CrCl ≥80 mL/min (1.6/100 patient-years; 95% CI: 1.1–2.2).

**Table 3:** Crude and standardized incidence rates of dabigatran patients stratified by creatinine clearance

	Crude incidence rates, per 100 patient-years (95% CI)			Standardized incidence rates, per 100 patient-years (95% CI)		
Event	CrCl ≥ 80 ( *n* = 1,738)	CrCl 50–79 ( *n* = 1,483)	CrCl 30–49 ( *n* = 475)	CrCl ≥ 80	CrCl 50–79	CrCl 30–49
Stroke						
Stroke a	0.53 (0.29–0.89)	0.96 (0.6–1.5)	0.46 (0.1–1.4)	0.58 (0.30–0.90)	0.85 (0.51–1.2)	0.33 (0.06–1.1)
Ischemic or hemorrhagic stroke	0.46 (0.24–0.8)	0.73 (0.42–1.2)	0.46 (0.1–1.4)	0.47 (0.23–0.75)	0.59 (0.33–0.88)	0.23 (0–0.53)
Death						
All cause	1.2 (0.83–1.7)	2.7 (2.1–3.5)	5.4 (3.8–7.5)	1.6 (1.1–2.2)	2.6 (2.0–3.3)	4.5 (3.1–6.3)
Vascular	0.49 (0.26–0.84)	0.78 (0.45–1.3)	1.7 (0.85–3.0)	0.69 (0.37–1.1)	0.81 (0.49–1.2)	1.8 (0.81–3.4)
Non-vascular	0.45 (0.23–0.79)	1.3 (0.85–1.9)	2.2 (1.2–3.6)	0.59 (0.30–0.96)	1.2 (0.74–1.6)	1.5 (0.77–2.3)
Major bleeding						
All major bleeding	0.72 (0.43–1.1)	1.1 (0.67–1.6)	2 (1.1–3.4)	0.68 (0.39–1.0)	0.92 (0.58–1.3)	1.26 (0.66–2.0)
Life-threatening	0.3 (0.13–0.6)	0.5 (0.25–0.9)	0.93 (0.34–2.0)	0.29 (0.11–0.53)	0.45 (0.22–0.72)	0.54 (0.19–1.0)

Abbreviations: CI, confidence interval; CrCl, creatinine clearance.

Note: Patients with CrCl <30 mL/min were omitted from this analysis due to low numbers. Standardized incidence rates were calculated with all missing HAS-BLED and CrCl values imputed.

a Includes hemorrhagic, ischemic strokes, and strokes of unknown origin.

## Discussion

The first main finding of this large global prospective study is that VKA prescription increased and dabigatran prescription decreased with declining renal function. No clear trend could be observed for the prescription of other NOACs and antiplatelet/no treatment groups. Second, less than 1% of dabigatran users were prescribed the contraindicated 150 and 110 mg twice-daily dosages when their CrCl was <30 mL/min. Third, among dabigatran users, the estimated rates of stroke and major bleeding were low across all CrCl categories.

Consistent with the EURObservational Research Program: the Heart Failure Pilot Survey on patients with AF (EORP-AF Pilot), nearly 60% of enrolled patients had a CrCl 30 to 79 mL/min and 4% had a CrCl <30 mL/min. 21 Patients with declining renal function were older and had a higher risk of stroke (CHA2DS2-VASc score ≥ 2) and bleeding risk (HAS-BLED ≥ 3). This might have led to a decrease in dabigatran prescriptions, although both the 150 and 110 mg twice daily doses have been found to be safe in patients with a CrCl 30 to 79 mL/min. 22 23 Conversely, VKA prescriptions increased, with the highest incidence of 47% in patients with a CrCl <30 mL/min. Consistent with other studies, the use of the dabigatran 150 and 110 mg twice daily doses in patients with a CrCl <30 mL/min was less than 1%, indicating that contraindicated dabigatran use is rare in clinical practice. 24 25 For other NOACs, similar prescription rates were observed across CrCl groups, which is probably related to label differences (i.e., <15 mL/min for rivaroxaban, edoxaban, and apixaban in the European Union, and no contraindications for rivaroxaban and apixaban in the United States). Moreover, the enrollment period must be taken into account. As dabigatran was the only NOAC approved in the European Union during the first year of study enrollment, higher number of dabigatran prescriptions are included in this assessed cohort as compared with other NOACs.

We found low rates of stroke (rate estimates range from 0.46 to 0.96/100 patient-years) and major bleeding (rate estimates range from 0.72 to 2/100 patient-years) across all CrCl categories with wide and overlapping confidence intervals. These rates are consistent with those observed in the EORP-AF Pilot registry in which the 1-year rates of stroke and major bleeding were similar across different renal groups. 21 However, although not directly comparable, the RE-LY trial reported increasing stroke, systemic embolism, and major bleeding incidences with declining renal function, with highest risk estimates in patients with a CrCL 30 to 49 mL/min and lowest in those with normal renal function, including patients using VKA. 23 26 Importantly, the RE-LY study was a randomized trial without specified dose reductions and thereby evaluated both dabigatran 150 and 110 mg twice daily doses, independent of CrCl. Conversely, in our study, dosing was individualized based on patient characteristics and physician and/or patient preferences, including the perceived bleeding risk. Therefore, our findings emphasize that dabigatran can safely be used in patients with mild-to-moderate impairment. Of note, in the ongoing phase III of GLORIA-AF follow-up data will be collected from VKA-treated patients, allowing for future comparison of safety and effectiveness. 13

### Limitations

This study has several limitations. First, CrCl values were missing in 21% of patients and were imputed for the outcome analyses. Second, due to broad inclusion of many centers and countries in this global registry, slight differences in NOAC labels existed regarding CrCl, which might have influenced the prescription pattern. Third, while we standardized for CHADS-VASc and HAS-BLED scores, other differences in baseline characteristics CrCl groups may not adequately have been captured. Finally, we assessed renal function at baseline and did not assess changes in renal function over the follow-up period.

In conclusion, in contrast to VKA, dabigatran prescription decreased with declining renal function and was rare in patients with severe renal impairment. Further, in dabigatran-treated patients, the estimated rates of stroke and major bleeding were low across the categories of renal impairment.
