# Supplementary material for: Anticoagulation Prescription and Outcomes in Relation to Renal Function in Patients with Atrial Fibrillation: Results from GLORIA-AF
Source: TH Open. 2021 Feb 6;5(1):e35–42. doi: 10.1055/s-0040-1722706 (PMC7867412; doi:10.1055/s-0040-1722706)
Supplement: Supplementary file 1 — Supplementary Material [file 10-1055-s-0040-1722706-s200095.pdf]

Supplementary Material

Phase II GLORIA AF Principal Investigators

Dzifa Abban
Nasser Abdul
Mark Abelson
Alan Ackermann
Fran Adams
Luthando Adams
Pedro Adragão
Walter Ageno
Rajesh Aggarwal
Sergio Agosti
Javier Aguila Marin
Francisco Aguilar
Julio Alberto Aguilar Linares
Luis Aguinaga
Zia Ahmad
Paul Ainsworth
Kamal Al Ghalayini
Saad Al Ismail
Abdelfatah Alasfar
Abdul Alawwa
Raed Al-Dallow
Lisa Alderson
Dimitrios Alexopoulos
Abdullah Ali
Malik Ali
Pareed Aliyar
Tammam Al-Joundi
Soufian Al Mahameed
Hossein Almassi
Khalid Almuti
Mohamed Al-Obaidi
Mohamed Alshehri
Ute Altmann
Alvaro Rabelo Alves Jr
Ayham Al-Zoebi
Walid Amara
Mathieu Amelot
Nima Amjadi
Fabrizio Ammirati
Nabil Andrawis
Denis Angoulvant
Giorgio Annoni
Gerardo Ansalone
Sorin Alexandru Antonescu
Mehrdad Ariani
Juan Carlos Arias
Sébastien Armero
Rohit Arora
Chander Arora
William Ashcraft
M. Shakil Aslam
Alfredo Astesiano
Philippe Audouin
Charles Augenbraun
S. Aydin
Rabih Azar
Abul Azim
Shahid Aziz
Luciano Marcelo Backes
Mirza Baig
Suchdeep Bains
Asaad Bakbak
Seth Baker
Karim Bakhtiar
Richard Bala
Jonathan Banayan
Stellan Bandh
Shigenobu Bando
Subhash Banerjee
Alan Bank
Olga Barbarash
Gonzalo Barón
Craig Barr
Carlos Barrera
John Barton
Vanja Basic Kes
Giovanni Baula
Hamid Bayeh
Nooshin Bazargani
Steffen Behrens
Alan Bell
Juan Benezet-Mazuecos
Bouziane Benhalima
Philippe Berdagué
B.J. Berg van den
P.F.M.M. Bergen van
Edvard Berngard
Richard Bernstein
Percy Berrospi
Sergio Berti
Vicente Bertomeu
Andrea Berz
Paulo Bettencourt
Robert Betzu
Jan Beyer-Westendorf
Ravi Bhagwat
Toby Black
Jorge Hugo Blanco Ibaceta
Stephen Bloom
Edwin Blumberg
Mario Bo
Valerie Bockisch

 © 2021. The Author(s).

Ellen Bøhmer
Maria Grazia Bongiorni
Giuseppe Boriani
Ralph Bosch
D.J. Boswijk
Jochen Bott
Edo Bottacchi
Marica Bracic Kalan
Axel Brandes
Bjørn Bratland
Donald Brautigam
Nicolas Breton
P.J.A.M. Brouwers
Kevin Browne
Jordi Bruguera
Myriam Brunehaut
Claude Brunschwig
Hervé Buathier
Aurélie Buhl
John Bullinga
Kenneth Butcher
Jose Walter Cabrera Honorio
Alberto Caccavo
Didier Cadinot
Shanglang Cai
Valeria Calvi
John Camm
Rui Candeias
James Capo Jr.
Alessandro Capucci
Juliano Novaes Cardoso
Yan Carlos Duarte Vera
Brian Carlson
Paula Carvalho
Susanna Cary
Rene Casanova
Gavino Casu
Simon Cattan
Claudio Cavallini
Guillaume Cayla
Tae Joon Cha
Kwang Soo Cha
Said Chaaban
Jei Keon Chae
Krishnan Challappa
Sunil Chand
Harinath Chandrashekar
Mark Chang
Paul Charbel
Ludovic Chartier
Kausik Chatterjee
Aamir Cheema
Shih-Ann Chen
Pierre Chevallereau
Fu-Tien Chiang
Francesco Chiarella
Lin Chih-Chan
Yong Keun Cho
Dong Ju Choi
Guy Chouinard
Danny, Hoi Fan Chow
Dimitrios Chrysos
Galina Chumakova
Eduardo Julián José Roberto Chuquiure Valenzuela
Tomas Cieza-Lara
Violeta Cindea Nica
Vlad Ciobotaru
David Cislowski
Olivier Citerne
Matthias Claus
Anthony Clay
Piers Clifford
Serge Cohen
Andrew Cohen
Furio Colivicchi
Rónán Collins
Steve Compton
Sean Connors
Alberto Conti
Gabriel Contreras Buenostro
Gregg Coodley
Martin Cooper
Lynn Corbett
Oran Corey
Julián Coronel
John Corrigan
Rosa Ysabel Cotrina Pereyra
Yves Cottin
Benoit Coutu
Aurel Cracan
Peter Crean
James Crenshaw
H.J.G.M. Crijns
Charles Crump
Fred Cucher
David Cudmore
Lianqun Cui
John Culp
Harald Darius
Patrick Dary
Olivier Dascotte
Ira Dauber
Thomas Davee
Ruth Davies
Gershan Davis
Jean-Marc Davy
Mark Dayer
Axel De La Briolle
Manuel de Mora
Eduardo De Teresa
Luc De Wolf
Eric Decoulx
Sasalu Deepak
Pascal Defaye

 © 2021. The Author(s).

Freddy Del-Carpio Munoz
Diana Delic Brkljacic
Laurent Deluche
Sylvain Destrac
N.Joseph Deumite
Silvia Di Legge
Olivier Dibon
Igor Diemberger
Jean Dillinger
Pedro Dionísio
Stefan Naydenov
Imran Dotani
Elena Dotcheva
Anthony D'Souza
Simon Dubrey
Xavier Ducrocq
Dmitry Dupljakov
Vuong DuThinh
Oscar Pereira Dutra
Dipankar Dutta
Nathalie Duvilla
Johnny Dy
Rainer Dziewas
Charles Eaton
William Eaves
Matthew Ebinger
J.W.M. Eck van
Tim Edwards
Isabel Egocheaga
Clifford Ehrlich
Steven Eisenberg
Abdel El Hallak
Adnan El Jabali
Rami El Mahmoud
Mahfouz El Shahawy
Zayd Eldadah
Fouad Elghelbazouri
Omar Elhag
Mehiar El-Hamdani
Darlene Elias
Adam Ellery
Hassan El-Sayed
A. Elvan
Bernard Erickson
Eric Espaliat
Louis Essandoh
Tamara Everington
Rudolph Evonich III
Andrey Ezhov
Lorenzo Fácila
Ramin Farsad
Maxime Fayard
Francesco Fedele
Luis Gustavo Gomes Ferreira
Daniel Ferreira
José Ferreira Santos
Anna Ferrier
Alexandra Finsen
Brian First
Raymond Fisher
John Floyd
Thomas Folk
Catarina Fonseca
Luisa Fonseca
Steven Forman
Magnus Forsgren
Malcolm Foster
Nathan Foster
Michael Frais
Brad Frandsen
Thierry Frappé
Ramon Freixa
William French
Marina Freydlin
Siegfried Frickel
Ana Gabriela Fruntelata
Shigeru Fujii
Yusuke Fujino
Hiroshi Fukunaga
Yutaka Furukawa
Matthias Gabelmann
Michael Gabris
Niels Gadsbøll
Pavel Galin
Michel Galinier
Ricky Ganim
Ronnie Garcia
Antonio Garcia Quintana
Olivier Gartenlaub
Conrad Genz
Frédéric Georger
Jean-Louis Georges
Steven Georgeson
Ali Ghanbasha
Evaldas Giedrimas
Mariusz Gierba
Eve Gillespie
Alberto Giniger
Alexandros Gkotsis
Joachim Gmehling
Jacek Gniot
Peter Goethals
Ronald Goldberg
Britta Goldmann
David Goldscher
Sergey Golitsyn
Efrain Alonso Gomez Lopez
Juan Esteban Gomez Mesa
Efrain Gonzalez
Emilio Gonzalez Cocina
Carlos Gonzalez Juanatey
Vladimir Gorbunov
Brian Gordon
Hervé Gorka

 © 2021. The Author(s).

Charles Gornick
Diana Gorog
Franz Goss
Andreas Götte
Pascal Goube
Ioannis Goudevenos
Dudley Goulden
Brett Graham
Angel Grande
Cesare Greco
Martin Green
Gerald Greer
Uwe Gremmler
Paul Grena
Yuriy Grinshstein
Martin Grond
Edoardo Gronda
Francois Grondin
Gerian Grönefeld
J.R. Groot de
Gabriele Guardigli
Thomas Guarnieri
Carolina Guevara Caiedo
Alexandre Guignier
Michele Gulizia
Michael Gumbley
Dhiraj Gupta
Terrence Hack
Winfried Haerer
Joseph Hakas
Christian Hall
James Hampsey
Georgios Hananis
Basel Hanbali
Franklin Handel
Joe Hargrove
David Hargroves
Kenneth Harris
David Hartley
Tetsuya Haruna
Yoshiki Hata
Emil Hayek
Jeff Healey
Steven Hearne
Geir Heggelund
M.E.W. Hemels
Yann Hemery
Sam Henein
Benhur Henz
Sung-Ho Her
Paul Hermany
Mauro Esteves Hernandes
Yorihiko Higashino
Michael Hill
Tetsuo Hisadome
Eiji Hishida
James Hitchcock
Etienne Hoffer
Matthew Hoghton
Clare Holmes
Suk Keun Hong
Marie-Paule Houppe Nousse
Victor Howard
Li Fern Hsu
Chi-Hung Huang
David Huckins
Kier Huehnergarth
A. Huizenga
Richard Huntley
Gamal Hussein
Gyo-Seung Hwang
Oyidie Igbokidi
Ignacio Iglesias
Margaret Ikpoh
Davide Imberti
Hüseyin Ince
Ciro Indolfi
Tatiana Ionova
John Ip
Didier Irles
Harukazu Iseki
Younus Ismail
Noah Israel
Steven Isserman
Bruce Iteld
Galina Ivanchura
Ramakrishnan Iyer
Venkat Iyer
Ruben Omar Iza Villanueva
Ewart Jackson-Voyzey
Naseem Jaffrani
Frank Jäger
Manish Jain
Martin James
Yann Jamon
Sung Won Jang
Cesar Augusto Pereira Jardim
Nabil Jarmukli
Robert Jeanfreau
Ronald Jenkins
Xianyan Jiang
Heng Jiang
Tiemin Jiang
Nan Jiang
Javier Jimenez
Robert Jobe
Ian Joffe
Bengt Johansson
Nick Jones
Jose Carlos Moura Jorge
Bernard Jouve
Mayar Jundi
Werner Jung
Byung Chun Jung

 © 2021. The Author(s).

Kyung Tae Jung
Samer Kabbani
Ameer Kabour
Chrystalenia Kafkala
Koji Kajiwara
Larisa Kalinina
Priit Kampus
Junji Kanda
Shaival Kapadia
Amin Karim
Laszlo Karolyi
Hisham Kashou
Andreas Kastrup
Apostolos Katsivas
Elizabeth Kaufman
Kazuya Kawai
Kenji Kawajiri
John Kazmierski
Phil Keeling
Galal Ali Kerfes
José Francisco Kerr Saraiva
Galina Ketova
Ajit Khaira
Muhammad Khalid
Elena Khludeeva
Aleksey Khripun
Doo Il Kim
Dae Kyeong Kim
Nam Ho Kim
Ki Seok Kim
Young-Hoon Kim
Jin bae Kim
June Soo Kim
Jeong Su Kim
Elena Kinova
Alexander Klein
Christoph Kleinschnitz
James Kmetzo
G. Larsen Kneller
Aleksandar Knezevic
Stanley Koch
Kai Koenig
Su Mei Angela Koh
Martin Köhrmann
Jay Koons
Ravikiran Korabathina
Olga Korennova
Martin Koschutnik
Edward Kosinski
Dragan Kovacic
Jacek Kowalczyk
Natalya Koziolova
J.A. Kragten
Lars Udo Krause
Imad Kreidieh
B.J. Krenning
Kannappan Krishnaswamy
Waldemar Krysiak
Karl-Heinz Kuck
Somnath Kumar
Thomas Kümler
Malte Kuniss
Jen-Yuan Kuo
Achim Küppers
Karla Kurrelmeyer
Tak Kwan
Eisho Kyo
Arthur Labovitz
Alain Lacroix
Andy Lam
Fernando Tomas Lanas Zanetti
Charles Landau
Giancarlo Landini
Wilfried Lang
Torben Bjerregaard Larsen
Volker Laske
Karine Lavandier
Nicki Law
Moon Hyoung Lee
Daniel Lee
Ana Leitão
Dominique Lejay
Malgorzata Lelonek
Radoslaw Lenarczyk
Patrick Leprince
Benoit Lequeux
Matthias Leschke
Nicolas Ley
Zicheng Li
Yansheng Li
Xiaodong Li
Zhanquan Li
Weihua Li
Jianqiu Liang
Ira Lieber
Michael Lillestol
Ramon Horacio Limon Rodriguez
Hailong Lin
Gregory Lip
Jennifer Litchfield
Zhitao Liu
Xuebo Liu
Yalin Liu
Feng Liu
Wenhui Liu
Guillermo Antonio Llamas Esperon
Jose Luis Llisterri
Ted Lo
Eric Lo
Jose Maria Lobos
Bernhard-Paul Lodde
Philippe Loiselet
José López-Sendón
Adalberto Menezes Lorga Filho

 © 2021. The Author(s).

Ido Lori
Ming Luo
Steven Lupovitch
Philippe Lyrer
Hamed M. Zuhairy
Changsheng Ma
Genshan Ma
Hong Ma
Irene Madariaga
Koji Maeno
Dominique Magnin
Shahid Mahmood
Karen Mahood
Gustavo Maid
Sumeet Mainigi
Konstantinos Makaritsis
José Arturo Maldonado Villalon
Rohit Malhotra
Amir Malik
Catherine Mallecourt
Rajiv Mallik
Rickey Manning
Athanasios Manolis
Ioannis Mantas
Fernando Gabriel Manzur Jattin
Niccolo' Marcionni
Francisco Marín
Antonio Martín Santana
Jorge Martinez
Luis Martinez
Petra Maskova
Norberto Matadamas Hernández
Simon Matskeplishvili
Katsuhiro Matsuda
Alenka Mavri
Erik May
Nolan Mayer
Pilar Mazon
John McClure
Terry McCormack
William McGarity
Michael McGuire
Hugh McIntyre
Paul McLaughlin
Brent McLaurin
Feliz Alvaro Medina Palomino
Paresh Mehta
Reza Mehzad
Andreas Meinel,
Francesco Melandri
Amparo Mena
Hirosi Meno
Dhananjai Menzies
Kneale Metcalf
Beat Meyer
Jacek Miarka
Frank Mibach
Dominik Michalski
Patrik Michel
Rami Mihail Chreih
Ghiath Mikdadi
Magdy Mikhail
Milan Mikus
Davor Milicic
Constantin Militaru
Gary Miller
Christos Milonas
Bogdan Minescu
Iveta Mintale
Aurélien Miralles
Tristan Mirault
Dinesh Mistry
George Mitchell
Nicoleta Violeta Miu
Naomasa Miyamoto
Tiziano Moccetti
Akber Mohammed
Azlisham Mohd Nor
Dora Ines Molina de Salazar
Giulio Molon
David Molony
Sergio Mondillo
Lluis Mont
Rajendra Moodley
Roger Moore
Dalmo Antonio Ribeiro Moreira
Kiyoo Mori
Andrew Moriarty
Jacek Morka
Nikitas Moschos
Marco Antônio Mota Gomes
Nicolas Mousallem
Angel Moya
Andreas Mügge
Thomas Mulhearn
Jean-Joseph Muller
Carmen Manuela Muresan
Derek Muse
Wlodzimierz Musial
Francesco Musumeci
Venkatesh Nadar
Thuraia Nageh
Priya Nair
Hidemitsu Nakagawa
Yuichiro Nakamura
Toru Nakayama
Ki-Byeong Nam
Dmitry Napalkov
Indira Natarajan
Hemal Nayak
Libor Nechvatal
James Neiman
Pamela Nerheim
Fernando Carvalho Neuenschwander

Kunihiro Nishida
Alexey Nizov
Tatiana Novikova
Salvatore Novo
Ewa Nowalany-Kozielska
Emmanuel Nsah
Juan Carlos Nunez Fragoso
Ole Nyvad
Manuel Odin de Los Rios Ibarra
Martin O'Donnell
Philip O'Donnell
Dong Jin Oh
Yong Seog Oh
Chia Theng Daniel Oh
Gilles O'Hara
Kostas Oikonomou
Juan Jose Olalla
Zoran Olivari
Richard Oliver
Christoforos Olympios
John Osborne
Joaquin Osca
Raed Osman
Abayomi Osunkoya
Benzy Padanilam
Elizaveta Panchenko
A. Shekhar Pandey
Angelo Amato Vicenzo de Paola
Alexander Paraschos
Herbert Pardell
Hyung Wook Park
Jong Sung Park
Ratika Parkash
Ian Parker
Eric Parrens
Robert Parris
Enrico Passamonti
Jaydutt Patel
Rajesh Patel
William H. Pentz
Viktor Persic
Francesco Perticone
Patrick Peters
Sanjiv Petkar
Luis Felipe Pezo
David Pham
Gérald Phan Cao Phai
Stephen Phlaum
Julien Pineau
Armando Pineda-Velez
Riccardo Pini
Arnold Pinter
Fausto Pinto
Salvatore Pirelli
Nediljko Pivac
Attilia Maria Pizzini
Darko Pocanic
Cristian Gheorghe Calin Podoleanu
Carisi Anne Polanczyk
Petr Polasek
Zdravka Poljakovic
Stewart Pollock
Jose Polo
James Poock
Holger Poppert
Yamile Porro
Antonio Pose
François Poulain
Jean-Ernst Poulard
Joe Pouzar
Petr Povolny
Domingo Pozzer
Athanasios Pras
Neeraj Prasad
Sébastien Prevot
Konstantin Protasov
Laurent Prunier
John Puleo
Maurice Pye
Fatma Qaddoura
Jean-Michel Quedillac
Dimitar Raev
Sidiqullah Rahimi
Arturo Raisaro
Bhola Rama
Nandkishore Ranadive
Katie Randall
Naresh Ranjith
Nuno Raposo
Haroon Rashid
Christa Raters
Ursula Rauch-Kroehnert
Thomas Rebane
Stefan Regner
Michael Renzi
Miguel Agustin Reyes Rocha
Shabbir Reza
Luigi Ria
Dimitrios Richter
Hans Rickli
Kyle Rickner
Werner Rieker
Fausto Rigo
Tomas Ripoll
Luiz Eduardo Fonteles Ritt
Douglas Roberts
Carlos Rodríguez Pascual
Ignacio Rodriguez Briones
Humberto Rodriguez Reyes
Marc Roelke
Mark Roman
Francesco Romeo
E. Ronner
Thomas Ronziere

 © 2021. The Author(s).

F.A. Rooyer
David Rosenbaum
Sherryn Roth
Nadezda Rozkova
Miroslav Rubacek
Frank Rubalcava
Olesya Rubanenko
Andrew Rubin
Mariano Ruiz Borret
Karin Rybak
Hani Sabbour
Oscar Saenz Morales
Tetsuo Sakai
Abraham Salacata
Ilsbe Salecker,
Adrien Salem
Marwan Salfity
Rafael Salguero
Alessandro Salvioni
Mercedes Samson
Gregorio Sanchez
Chirag Sandesara
Wladmir Faustino Saporito
Taishi Sasaoka
Payman Sattar
Daniel Savard
Pierre-Jean Scala
Jacques Scemama
Thierry Schaupp
Peter Schellinger
Carlos Scherr
Karl-Heinz Schmitz
Bettina Schmitz
Lisa Schmitz
Robert Schnitzler
Steffen Schnupp
Peter Schoeniger
Norbert Schön
Stefan Schuster
Peter Schwimmbeck
Clare Seamark
Ruediger Seebass
Karl-Heinz Seidl
Barry Seidman
Jaroslaw Sek
Lakshmanan Sekaran
Yoshinori Seko
Pablo Andres Sepulveda Varela
Begoña Sevilla
Vinay Shah
Anil Shah
Neerav Shah
Aman Shah
Jeffrey Shanes
Ali Sharareh
Vijay Kumar Sharma
Louise Shaw
Yutaka Shimizu
Hideki Shimomura
Dong Gu Shin
Eun-Seok Shin
Junya Shite
Mohammad Shoukfeh
Charles Shoultz III
Frank Silver
Iveta Sime
T.A. Simmers
Dinesh Singal
Narendra Singh
Peter Siostrzonek
Mohiburrahman Sirajuddin
Mika Skeppholm
Didier Smadja
Richard Smith
David Smith
Hassan Soda
C. Wilson Sofley, Jr.
Adam Sokal
Rodolfo Sotolongo
Olga Ferreira de Souza
Jon Arne Sparby
Jindrich Spinar
David Sprigings
Alex Spyropoulos
Dimitrios Stakos
Alon Steinberg
Clemens Steinwender
Georgios Stergiou
H. William Stites
Anastas Stoikov
Ruth Strasser
Witold Streb
Ioannis Styliadis
Guohai Su
Xi Su
Rafael Martin Suarez
Wanda Sudnik
Atsushi Sueyoshi
Kai Sukles
Li Sun
Randeep Suneja
Peter Svensson
Antonius Ziekenhuis
Janko Szavits-Nossan
Jens Taggeselle
Yuichiro Takagi
Amrit Takhar
Julio Tallet
Angelika Tamm
Shozo Tanaka
Katsumi Tanaka
Aylmer Tang
Sherman Tang
Tiziana Tassinari

 © 2021. The Author(s).

Shinji Tayama
Muzahir Tayebjee
Ulrich Tebbe
Jose Teixeira
Dan Nicolae Tesloianu
Pascal Tessier
S.H.K. The
Jérome Thevenin
Harold Thomas
Serge Timsit
Robert Topkis
Mikhail Torosoff
Emmanuel Touze
Thalie Traissac
Elina Trendafilova
Barry Troyan
Wenchi Kevin Tsai
Hung Fat Tse
Hiroshi Tsutsui
Takashi Tsutsui
Y.S. Tuininga
Minang Turakhia
Samir Turk
Wayne Turner
Arnljot Tveit
Shannon Twiddy
Richard Tytus
Gerald Ukrainski
Salvador Bruno Valdovinos Chavez
Eric Van De Graaff
Peter Vanacker
Panagiotis Vardas
Michael Vargas
Vassilios Vassilikos
Juan Vazquez
Asok Venkataraman
Paolo Verdecchia
Ernst Günter Vester
Hubert Vial
Dragos Vinereanu
Anthony Vlastaris
Craig Vogel
Jürgen vom Dahl
Matthias von Mering
Kishor Vora
Paul Wakefield
Jasjit Walia
Thomas Walter
Mingsheng Wang
Ningfu Wang
Feng Wang
Xinhua Wang
Zulu Wang
Kuo-Yang Wang
Kouki Watanabe
Jeanne Wei
Christian Weimar
Renate Weinrich
Ming-Shien Wen
Kevin Wheelan
Jens Wicke
Marcus Wiemer
Beate Wild
Andreas Wilke
Stephan Willems
Marcus Williams
David Williams
Andreas Winkler
Jost Henner Wirtz
Bernhard Witzenbichler
Danny H K Wong
Ka Sing Lawrence Wong
Brian Wong
Beata Wozakowska-Kaplon
Zhaohui Wu
Shulin Wu
Nell Wyatt
Yong Xu
Xiangdong Xu
Akira Yamada
Kazuya Yamamoto
Hiroki Yamanoue
Takeshi Yamashita
Ping Yen Bryan Yan
Yanmin Yang
Tianlun Yang
Jing Yao
Chakri Yarlagadda
Kuo-Ho Yeh
Yoto Yotov
Serge Yvorra
Ralf Zahn
José Zamorano
Roberto Zanini
Stuart Zarich
James Zebrack
Sergei Zenin
Elisabeth Louise Zeuthen
Xingwei Zhang
Quansan Zhang
Dadong Zhang
Donghui Zhang
Huanyi Zhang
Shuiping Zhao
Xinwen Zhao
Yang Zheng
Qiangsun Zheng
Jing Zhou
Jian Zhou
Sergio Luiz Zimmermann
Rainer Zimmermann
L. Steven Zukerman
C. Zwaan van der

 © 2021. The Author(s).
